# Transitional nystagmus in a Bow Hunter’s Syndrome case report

**DOI:** 10.1186/s12883-020-02009-3

**Published:** 2020-11-30

**Authors:** Yasuyuki Nomura, Teruo Toi, Yasuo Ogawa, Takeshi Oshima, Yuichiro Saito

**Affiliations:** 1 Department of Otolaryngology - Head and Neck Surgery, Nihon University School of Medicine, 30-1, Oyaguchi-kamicho, Itabashi-ku, Tokyo, 173-8610 Japan; 2 Department of Otorhinolaryngology, Tokyo Medical University, Hachioji Medical Center, 1163 Tatemachi, Hachioji-city, Tokyo, 193-0998 Japan; 3 Saito Clinic, 5-20-11, Sakurajosui, Setagaya-ku, Tokyo, 156-0045 Japan

**Keywords:** Bow Hunter’s syndrome, Vertigo, Nystagmus, Vertebral artery, Case report

## Abstract

**Background:**

Bow Hunter’s Syndrome (BHS) is known as one of cervical diseases which causes vertigo, but the details of its vertigo, especially nystagmus and eye movement, are still incompletely understood. This time, we reported the first case of BHS with a nystagmus chart with video record of transitional nystagmus.

**Case presentation:**

The patient, a 47-year-old female, complained of vertigo caused by head rotation. When she turned her head leftward, leftward nystagmus appeared, and this was followed by dullness of the right arm. After her head was returned to the central position, downbeat nystagmus appeared, which changed to rightward nystagmus. She was diagnosed with BHS by her symptoms and images. We recorded a nystagmus video and nystagmus chart of this transitional nystagmus including downbeat nystagmus. Her vertigo was cured by the modification of a prescription for her past medical history: hypertension.

**Conclusion:**

The vertigo of BHS accompanies nystagmus. In this present case, the transitional nystagmus was observed, and it occurred toward the healthy side. Then the nystagmus direction was changed to the affected side via downbeat nystagmus. This is the first report with both a nystagmus chart with video of BHS. Nowadays, various kinds of vertigo induced by neck movement are known. BHS is a rare disease among vertigo diseases, but we should consider it as a different diagnosis of vertigo patients. A precise interview and proper examination are required to make the final diagnosis.

## Background

Bow Hunter’s Syndrome (BHS) is known as one of cervical diseases which causes vertigo, but the details of its vertigo, especially nystagmus and eye movement, are still incompletely understood. In 1978, Sorensen reported a brain stem infarction observed during archery training and called it bow hunter’s stroke [1]. Thereafter, BHS was defined as a disease in which ischemic symptoms developed due to the reduction of blood flow of the vertebral artery (VA) at a time of rotating the neck similar to when shooting a bow [2, 3]. Reviewing previous studies of BHS, we compiled many reports of the BHS cases involved vertigo [4–8].

However, the descriptions of detailed nystagmus were quite few. Until now, there has been no report that has shown a nystagmus chart of BHS. This time, we reported the first case of BHS with a nystagmus chart with video record. In addition, the nystagmus of this case: transitional nystagmus accompanied with downbeat nystagmus, has not been reported for BHS so far. It might be helpful to research nystagmus and vertigo induced by ischemia. We should consider BHS as a differential diagnosis of vertigo, which is induced by neck movement such as benign paroxysmal positional vertigo (BPPV).

## Case presentation

A 47-year-old female complained of vertigo caused by head rotation. Approximately 2 months prior to visiting a neighborhood clinic, vertigo, nausea and dullness of the right arm had begun when she turned her face to the left. Bow Hunter’s Syndrome (BHS) was suspected at that clinic and she was referred to our hospital for scrutiny. Medical history: she had been prescribed antihypertensive agents. She and her family did not have any genetic past history.

There were no abnormalities concerning neurological examinations and she did not reveal any dysmetria at her initial visit to our hospital. However, when she turned her head leftward for approximately 15 s, leftward nystagmus appeared, and this was followed by dullness of the right arm. Approximately 25 s after the onset of dullness, the patient complained of severe nausea. After the head was returned to the central position, downbeat nystagmus appeared, which changed to rightward nystagmus, then disappeared after 30 s. These transitional nystagmus and symptoms were observed both in the sitting and supine positions.

**Fig. 1:**
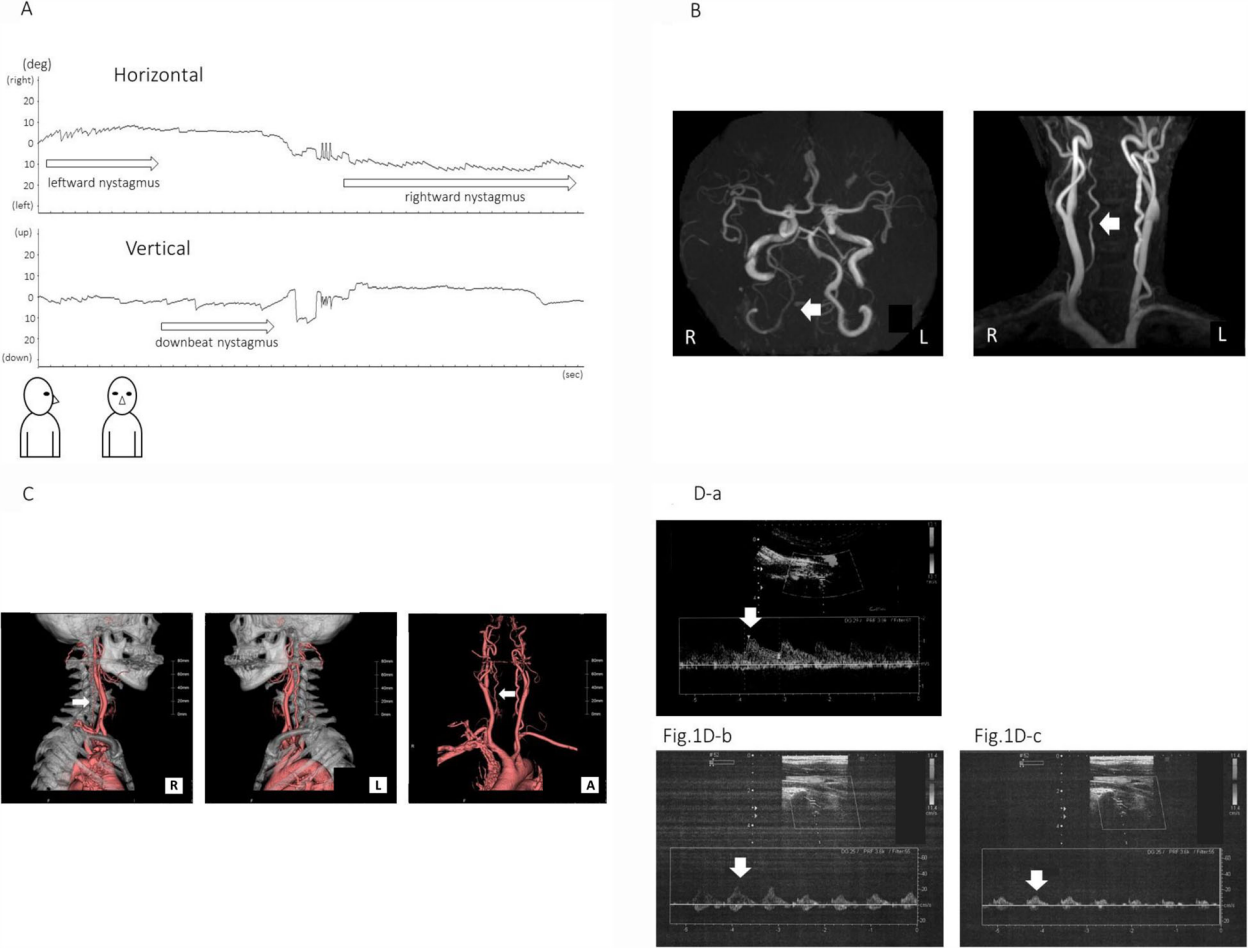
**A** The nystagmus at her second visit in the sitting position; the leftward nystagmus according to the leftward neck rotation transited to the rightward nystagmus via downbeat nystagmus after the head returned to the center position. The nystagmus was analyzed by the infrared CCD camera using a macro program in Image J (National Institutes of Health, USA), which was provided by the Department of Otolaryngology, Yamaguchi University, Japan. **B** MR angiography showed the hypoplasia of the right vertebral artery in intracranial and extracranial portions. **C** 3D-CT also showed the hypoplasia of the right vertebral artery (white arrows). **D** Carotid ultrasonography in supine position: The blood flow of the right vertebral artery (Fig. 1D-a) was less than the left (Fig. 1D-b) even in the supine position. Then, when the neck was rotated leftward, further reduction of blood flow was seen in the doppler waveform of the right vertebral artery (Fig. 1C-c) (white arrows)


Her nystagmus at her first visit was unrecordable, therefore Fig. 1A (and Additional file video) shows the nystagmus at her second visit in a sitting position using infrared CCD video-oculography.

**Additional file 1.**

The routine electronystagmography and equilibrium tests were normal. MRI showed no abnormal findings in the brain, but MR Angiography and 3D-CT detected right vertebral artery (VA) hypoplasia (Fig. 1B, C) and carotid ultrasonography showed the reduction of blood flow of the right VA. (Fig. 1D). There was no bony deformation which was responsible for pressing on the right VA with head rotation on CT. “In addition, there were no symptoms or reduction in flow in the dominant left vertebral artery on turning the head to the contralateral side. We could not record the nystagmus and the carotid doppler concurrently.

According to these symptoms and findings, BHS was diagnosed, and we consulted with the neurosurgery and orthopedic surgery departments. Conservative therapy was advised and she was told to avoid rotating her neck leftward in her daily life.

Afterwards, she noticed that her systolic blood pressure was too low, i.e., 80–90 mmHg every evening under the medication for her hypertension. At last, the prescription modified by a new physician elevated her blood pressure and the vertigo symptoms also improved. Her systolic blood pressure after her hypertensive medication was stopped was about 120–130 mmHg. She has not suffered a vertigo attack since.

## Discussion and conclusion

BHS is known as a disease which reveals vertigo according to the patient’s cervical movement especially lateral convolution. It is caused by ischemia developed due to the reduction of blood flow of VA at a time of rotating the neck [2, 3]. As we described above, when we review previous studies of BHS, we compiled many reports of the BHS cases involved vertigo [4–8]. However, the descriptions of detailed nystagmus were quite few. Iida et al. [5] described a case report with the video of downbeat nystagmus. They also reported that nine of twenty BHS cases had vertigo symptom in their literature review. Ogino et al. [7] mentioned that vertical nystagmus appeared in their case. Matsuyama et al. [8] reported a case with nystagmus toward the opposite direction for the neck rotation. The reason why so few reports described the detailed eye movements or nystagmus is that it is too difficult to observe precisely and record them during the patient’s vertigo attack. Therefore, there has been no report that has shown a nystagmus chart with video of BHS. This time, we reported the first case of BHS with a nystagmus chart with video record. In addition to that, this report has another importance: the affected side and healthy side of the VA were cleared by doppler echo and images. By this, the direction of nystagmus towards the affected side and healthy side were also detected.

In this present case, leftward rotation of the neck led to the appearance of leftward nystagmus. Returning the neck to the head-center position led to a change to rightward nystagmus via downbeat nystagmus. It was supposed that the ischemia of the brain was caused by the deficiency of the blood flow of the right VA according to the imaging and doppler echo findings. Considering the mechanism of this transitional nystagmus, the ischemia of the right VA caused the nystagmus towards the healthy left side. This was caused by the reduction of the right cerebellum or brainstem inhibition. This kind of nystagmus towards the healthy side is sometimes observed among patients with vertigo caused by verteblobasilar artery insufficiency. In this case, according to the recovery of the blood flow when the neck rotation was reset to the center position, rightward nystagmus was observed. We supposed that this rightward nystagmus was caused by the alternative change of the relative relation between the right and left central vestibular system, such as the vestibular nuclei of both sides and the cerebellum. Interestingly, the nystagmus observed between these two horizontal directions was downbeat nystagmus. In this case, the patient’s characteristic transitional nystagmus accompanied with downbeat nystagmus helped clarify the diagnosis of BHS. This downbeat nystagmus occurs just halfway through the transitional nystagmus from the direction of the healthy side to afferent side. The effect of ischemia or recovery course of brainstem might relate to this phenomenon, not upward but downbeat nystagmus. Previous reports by Ogino, et al. [7] and Matsuyama, et al. [8] might see just one phase of these kinds of changing nystagmus. Iida et al. [5] indicated that the downbeat nystagmus of their case was induced by the ischemic disfunction of the central vestibular area.

In terms of the cause of BHS, three different pathologies have been indicated. One is the case where the VA is compressed at the C1–2 level of the afferent side [4]. The second is the case developed due to neck rotation in the ipsilateral direction of the compressed vessel [9]. Our case is consistent with the latter. Another possible mechanism is that blood stagnation and thrombus formation occur at the site of stenosis of the VA, causing an embolism, as has been observed while maintaining neck rotation during surgery under general anesthesia [10].

Diagnosis of BHS is conventionally based on angiographic identification. However, in recent years, BHS diagnosis based on ultrasonography has become commonplace [11]. In our case, MRA and 3D-CT detected hypoplasia of the right VA and carotid ultrasonography detected a reduction in blood flow. However, before the imaging examinations, the primary step to make a proper diagnosis of BHS must be a detailed and precise interview with a careful examination in relation to nystagmus.

Reported treatments for BHS include both conservative and surgical intervention such as posterior cervical spinal fusion, decompression and stent placement [3, 5, 6]. To date, there is no consensus concerning the ideal therapy for BHS. It is supposed that there is a limitation to the treatment of BHS. The present case also required no surgical treatment, as dose reduction of antihypertensive agents led to an improvement of the vertigo symptoms.

As a take-away lesson, nowadays, various kinds of vertigo induced by neck movement are known. The most common vertigo disease relating to neck movement is perhaps BPPV. However, we should remember that vertigo is caused by ischemia accompanied by neck rotation like BHS. BHS is a rare disease among vertigo diseases, but we should consider it as a different diagnosis of vertigo patients. The precise interview and proper examination are required to make the final diagnosis. We believe a big clue to diagnose vertigo disease is to listen to the patients’ interviews very carefully, because only the patient knows the situation of their vertigo when it occurs.

The patient in this case could not have her vertigo diagnosed for a long time and visited several clinics. When we gave her the diagnosis of BHS with our definitive examination findings, she was relieved from her anxiety and could move forward with curing her symptoms. The knowledge of this disease and the clues that lead to diagnosis are also important to relieve patients.

## Data Availability

The authors declare that all the data are contained within the manuscript.

## Abbreviations

BHS: Bow Hunter’s Syndrome
VA: Vertebral artery
